# From Break-Even Point to Dynamic Regenerative Balance: A Conceptual and Quantitative Framework Based on Preclinical Rabbit Sinus Lift Data

**DOI:** 10.3390/dj13100469

**Published:** 2025-10-15

**Authors:** Daniele Botticelli, Karol Alí Apaza Alccayhuaman, Samuel Porfirio Xavier, Erick Ricardo Silva, Yasushi Nakajima, Shunsuke Baba

**Affiliations:** 1 Department of Oral Implantology, School of Dentistry, Osaka Dental University, 8-1 Kuzuhahanazonocho, Hirakata 573-1121, Osaka, Japan; y.nakajima@me.com (Y.N.); baba-s@cc.osaka-dent.ac.jp (S.B.); 2 ARDEC Academy, 47923 Rimini, Italy; karol.ali.apazaa@gmail.com; 3 Department of Oral Biology, University Clinic of Dentistry, Medical University of Vienna, 1090 Vienna, Austria; 4 Department of Oral and Maxillofacial Surgery and Periodontology, Faculty of Dentistry of Ribeirão Preto, University of São Paulo, Ribeirão Preto 14040-904, SP, Brazil; spx@forp.usp.br (S.P.X.); erick.silva@usp.br (E.R.S.)

**Keywords:** bone regeneration, osseointegration, biomaterials, histomorphometry, sinus augmentation, bone resorption, break-even point, Dynamic Regenerative Balance

## Abstract

**Background:** Traditional parameters such as bone-to-implant contact percentage (BIC%) provide only static insights into implant integration and do not reflect the temporal dynamics of bone regeneration. The concept of Dynamic Regenerative Balance (DRB) was introduced to represent the biological equilibrium between bone formation and graft resorption. The break-even point serves as a measurable approximation of this equilibrium. This study aimed to illustrate the usefulness of the break-even point in expressing the balance between graft resorption and new bone formation, rather than to define definitive values for specific biomaterials. **Methods**: Four preclinical studies on sinus floor elevation in rabbits were selected. Each reported histomorphometric data on new bone formation and graft resorption at two or more time points. Six biomaterials were analyzed: autogenous bone, Bio-Oss^®^, Bio-Oss Collagen^®^, Gen-Os^®^, Maxresorb^®^, and Maxresorb^®^ Inject. The break-even point was calculated by linear extrapolation as the time at which new bone equals residual graft percentage. **Results**: The break-even point varied significantly among biomaterials (expressed in days/area %): autogenous bone reached equilibrium fastest (18.4 days/13.5%), followed by Gen-Os^®^ (40.4 d/19.1%). Bio-Oss Collagen^®^ (62.3 d/28.3%), Maxresorb^®^ (73.9 d/36.4%), and Maxresorb^®^ Inject (96.1 d/34.1%). For Bio-Oss^®^, it occurred at 81.8 days (33.6%) in one study, while in another, it was not reached within 6 months. These differences reflect distinct regenerative kinetics and resorption profiles among materials. **Conclusions**: The break-even point offers a simple and informative parameter to describe the balance between graft resorption and new bone formation, providing a useful complement to conventional histomorphometric measures and a framework for future studies.

## 1. Introduction

Osteointegration is traditionally defined as the direct structural and functional connection between vital bone and the surface of a load-bearing implant [1]. As a biological concept, it encompasses the qualitative nature of the interface that is characterized by the absence of interposed fibrous tissue and the long-term mechanical stability achieved through bone anchorage. Osteointegration is not merely the physical apposition of bone to an implant surface; rather, it represents a comprehensive biological concept that incorporates the entire cascade of events leading to the stable and functional anchorage of an implant within living bone. This concept includes, even though not explicitly described, the myriad of molecular, cellular, and tissue-level phenomena that unfold during healing. These phenomena encompass clot formation, inflammatory response, angiogenesis, osteoblast differentiation, matrix deposition, and bone modeling and remodeling [2,3,4,5]. It has been shown that commercially pure titanium produces a foreign body reaction when placed in live tissues, a fact that actively influences osseointegration [6,7,8,9]. Simply put, osteointegration is the final outcome of a complex and multistage healing process and serves as a concept that integrates all underlying mechanisms regardless of their specific sequence, timing, or molecular pathways. Yet, despite its functional and clinical relevance, this concept remains difficult to quantify directly.

To bridge this gap, histomorphometric studies commonly employ the bone-to-implant contact percentage (BIC%) as a surrogate parameter to represent osteointegration [10,11]. BIC% provides a measurable estimate of the extent to which mineralized tissue is in direct contact with the implant surface in a two-dimensional histological section. While BIC% does express the degree of osteointegration in a specific location and at a specific time point, it does not explain how osteointegration occurred, nor does it capture the temporal dynamics or the quality of the newly formed bone. Thus, BIC% can be understood as a proxy indicator of osteointegration such as an attempt to translate a broad biological concept into a quantifiable metric. While widely adopted, this parameter presents several important limitations. First, it does not distinguish between newly formed and pre-existing bone, thereby failing to reflect the regenerative contribution of the healing process itself [12,13,14,15]. Second, it lacks spatial resolution in three dimensions and is inherently limited to two-dimensional histological sections, while microCT offers a 3-dimensional view [16]. Additionally, BIC% is highly sensitive to experimental variables such as species, bone quality, implant design, surface characteristics, site preparation and healing time, which limits its reproducibility and comparability across studies [17,18,19,20,21]. In addition, no universally accepted BIC% threshold exists to define clinical success, and absolute values may not be transferable across models or anatomical sites.

Also, being a percentage, BIC% does not account for the absolute extent of bone-implant contact. Two implants with identical BIC% may differ substantially in their total contact area depending on their geometry; a longer or wider implant will inherently provide more surface for integration than a shorter or narrower one. Consequently, BIC% alone does not represent the true mechanical relevance of osseointegration.

Finally, the term bone-to-implant contact may be misleading. It does not differentiate between mineralized bone and other osseous structures such as osteon canals, marrow spaces, or remodeling units (BMUs), which are histologically part of bone but do not contribute to direct mechanical support. Thus, a more precise terminology, such as mineralized bone-to-implant contact (MBIC), may better reflect the structural and functional relevance of the interface.

Another parameter should be considered besides BIC%, and it is related to the bone density around implants. The term bone quality is frequently used in both clinical and experimental literature, often without a precise definition [22,23,24]. It is commonly assumed that denser bone equates to better outcomes, while lower-density bone is considered suboptimal. Yet, this interpretation may be reductive. Optimal bone quality does not necessarily correspond to maximum mineral density, but rather to a balanced structural composition that allows for effective load distribution, biological turnover, and mechanical resilience.

Importantly, a high BIC%, even approaching complete bone coverage of the implant surface, does not guarantee mechanical stability if the surrounding bone volume is insufficient or poorly organized. Simply put, the structural integrity and functional performance of the peri-implant bone cannot be inferred from BIC% alone. A useful analogy can be drawn from civil engineering: an implant may be perfectly coated with mineralized tissue, much like a steel column encased in concrete (i.e., 100% BIC), but if that column is supported by only a few thin pillars (representing low bone density or weak trabecular structure), the entire system may fail when subjected to load. Conversely, a moderately high BIC% surrounded by a dense, well-oriented trabecular network may provide greater long-term support.

This shows the necessity of integrating both interface-level and volume-level parameters when evaluating implant integration. While BIC% offers insight into the direct contact at the implant surface, parameters such as bone area/volume fraction and overall bone density are essential to assess the mechanical competence of the surrounding tissue.

The break-even point, also referred to as the interception point in previous work [25], serves as a quantifiable approximation of what we define as the Dynamic Regenerative Balance (DRB), a theoretical moment in which newly formed bone equals resorbed graft, reflecting the biological turnover equilibrium.

The break-even point is defined as the time and value at that the proportion of newly formed bone equals that of resorbed pre-existing tissue, whether native bone or graft material, and the break-even point reflects the moment of biological equilibrium in the regeneration process.

This parameter provides added value by integrating spatial and temporal dimensions. It allows comparisons across species, implant surfaces, and biomaterials, and may help identify differences in regenerative dynamics that are invisible to conventional static metrics. For example, two implant surfaces may present similar BIC% at a given time point, yet exhibit very different rates of bone apposition, a difference that the break-even point can reveal [25].

We have previously applied this parameter to investigate the dynamics of osseointegration and peri-implant bone density in various preclinical models, including humans, dogs, and rabbits [25,26]. Our studies demonstrated that the break-even point is strongly influenced by species, implant surface, bone quality (e.g., cortical vs. trabecular), implant loading protocols (e.g., immediate or early loading), and anatomical conditions (e.g., immediate implant into the extraction socket). In sinus lift procedures, we observed distinct regenerative kinetics in regions adjacent to the sinus walls compared to areas located directly under the sinus mucosa [27].

These findings highlight the break-even point’s capacity to capture local variations in healing behavior that traditional metrics fail to detect. As such, the break-even point is not a replacement for BIC% or bone density metrics, but rather a complementary tool that enriches our understanding of implant integration through a time-based, biologically grounded perspective.

If applied to grafted sites, the break-even point might reflect the point at which the scaffold has been effectively replaced by vital bone, thus serving as a potential indicator of functional maturation.

In this study, we extend the application of the break-even point to analyze the relationship between new bone formation and graft resorption. By tracking the time at which the newly formed bone overtakes the residual graft, we aim to establish a quantitative benchmark that can support material selection in regenerative procedures. This approach may help identify graft materials that not only support early bone formation but also resorb in synchrony with tissue regeneration, an essential requirement for long-term success. Therefore, the aim of this study was not to determine definitive break-even point values for specific biomaterials, but rather to demonstrate the applicability of this parameter to express the relationship between graft resorption and new bone formation. By using data extracted from a limited number of available preclinical studies, the present analysis sought to provide a methodological and conceptual framework, rather than an exhaustive or statistically representative comparison among materials.

## 2. Materials and Methods

### 2.1. Selection of the Articles

A series of four articles on sinus floor elevation in rabbits that reported data on new bone formation and graft resorption rate in at least two different periods of healing were selected for analysis. Most of the articles were selected from the same research group and the same histological laboratory.

The following articles we selected:

Lambert et al. (2011) [28]: This study used clot or autogenous bone chips or deproteinized bovine bone mineral (Bio-Oss^®^ spongiosa granules 0.125–1.0 mm; Geistlich Pharma AG, Wolhusen, Switzerland). The examination periods were 1 week, 5 weeks and 6 months. The histological slides represented sagittal planes through the central portion of the sinus, corresponding to the area most distant from the bony walls.

Yamada et al. (2025) [29]: This study used deproteinized bovine bone mineral alone (Bio-Oss^®^ spongiosa granules 0.125–1.0 mm; Geistlich Pharma AG, Wolhusen, Switzerland) or in combination with 10% porcine-derived type I collagen (Bio-Oss Collagen^®^; Geistlich Pharma AG, Wolhusen, Switzerland). The examination periods were 2 and 12 weeks, and coronal sections were analyzed.

Costa et al. (2021) [30]: This study used synthetic biomaterial composed of a combination of hydroxyapatite (HA) and beta-tricalcium-phosphate (β-TCP), in proportions 60:40, respectively. The sinuses were filled with either a granular formulation (Maxresorb^®^, Botiss Biomaterials, Zossen, Germany) or a paste formulation (Maxresorb^®^ Inject, Botiss Biomaterials, Zossen, Germany). The examination periods were 2 and 10 weeks, and coronal sections were analyzed.

Iida T et al. (2017) [31]: This study used collagenic cortico-cancellous porcine bone (OsteoBiol^®^ Gen-Os^®^, Tecnoss^®^, Giaveno, Italy). Only the control sinuses were used, the ones with no collagen membrane placed underneath the sinus mucosa. The examination periods were 2, 4 and 8 weeks. The examination periods were 2 and 12 weeks, and coronal sections were analyzed.

### 2.2. Biomaterials Evaluated

Bio-Oss^®^ (Geistlich Pharma AG, Wolhusen, Switzerland) is a xenogeneic bone graft material of bovine origin, known for its osteoconductive properties and slow resorption rate. It is produced by removing organic components through a low-temperature deproteinization process at around 300 °C, which preserves its trabecular structure. The resulting material is highly porous and chemically similar to human cancellous bone [32].

Bio-Oss Collagen^®^ (Geistlich Pharma AG, Wolhusen, Switzerland), from the same manufacturer, combines Bio-Oss granules with 10% porcine type I collagen. The addition of collagen, which is naturally resorbed by the body, is designed to enhance the material’s cohesion and facilitate placement in the surgical site, particularly during the initial healing period.

Maxresorb^®^ (Botiss Biomaterials, Zossen, Germany) is an alloplastic bone substitute available in granular or injectable paste formulations. The granular version contains a biphasic ceramic blend of hydroxyapatite (HA) and beta-tricalcium phosphate (β-TCP) in a 60:40 ratio. It features a high overall porosity (around 80%) and includes interconnected macropores ranging from 200 to 800 µm [33].

Maxresorb^®^ Inject (Botiss Biomaterials, Zossen, Germany) is a ready-to-use formulation consisting of a hydrogel matrix incorporating HA nanoparticles along with fine HA and β-TCP granules in the same 60:40 proportion. The HA nanoparticles, measuring approximately 15–50 nm, contribute to a large surface area, promoting enhanced cellular attachment and interaction. The gel carrier ensures controlled resorption and improved handling properties during application [33].

OsteoBiol^®^ Gen-Os^®^ (Tecnoss^®^, Giaveno, Italy) is composed of collagenic corticocancellous bone obtained, in the analyzed study, from swine at temperatures up to 130 °C. The total and intraparticle porosities are 33.1% and 21%, respectively. The real density was 2.43 g/cm^3^ and the mineral content was 64.6% [32].

### 2.3. Break-Even Point Calculation

The concept of the break-even point refers to the moment in time when the percentage of newly formed bone equals that of resorbed graft. This point corresponds to the intersection between two trend lines: an ascending one representing new bone formation and a descending one indicating the resorption of the graft. It is characterized by two coordinates: the time at which it occurs (x, in days) and the corresponding percentage values of new bone and graft (y, in %). The break-even point is determined by solving a simple linear equation that identifies the intersection between these two lines. A more detailed explanation of the mathematical process used to identify this intersection is available in separate publications [25,26].

To identify the break-even point, it is necessary to obtain data at two different time intervals. Assuming linear progression between the time points, straight lines can be drawn and their intersection estimated. In cases with multiple available time points, the two closest to the anticipated crossing should be selected to ensure higher accuracy. All calculations were carried out using Microsoft Excel for Microsoft 365 (Microsoft Corporation, Redmond, WA, USA). The break-even point calculator is available in the Supplementary Material of this article (S1). Yet, artificial intelligence can perform the calculation just by providing data at two different time points. The graphs were obtained using PowerPoint for Microsoft 365 (Microsoft Corporation, Redmond, WA, USA).

### 2.4. Risk of Bias Assessment

Risk of bias assessment. The methodological quality of the included animal studies was assessed using the SYRCLE’s risk of bias (RoB) tool, which is specifically designed for preclinical research. Two reviewers performed the assessment independently (D.B. and K.A.A.A.A), and disagreements were resolved by discussion.

## 3. Results

Table 1 summarizes the key characteristics of the included studies, detailing the type of graft material, the healing periods analyzed, and the respective percentages of new bone formation and residual graft. These data, collected at two distinct timepoints (T1 and T2), provide the basis for comparing the temporal behavior of each biomaterial in terms of regenerative performance.

### 3.1. Autogenous Bone Chips

At 1 and 5 weeks, the mean percentage of newly formed bone was 0.3% and 32.7%, respectively, whereas the residual graft decreased from 22.7% to 0.0% (Table 1) [28]. These data indicate a highly dynamic remodeling process with near-complete graft resorption by 5 weeks.

**Figure 1:**
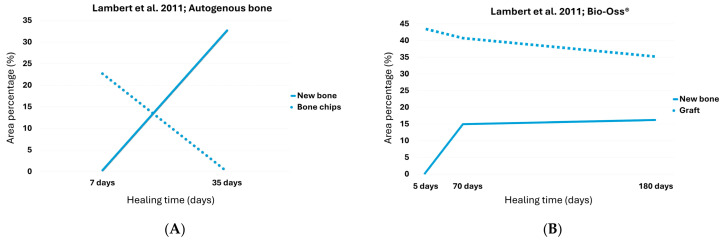
The point at which the two lines intersect represents the break-even point for (**A**) autogenous bone graft, (**B**) Bio-Oss^®^ [28].

Based on linear extrapolation between these two time points, the break-even point, defined as the point at that newly formed bone equals residual graft, was estimated to occur at approximately 18.4 days, with both components reaching approximately 13.5% total evaluated tissue (Figure 1A; Table 2).

Notably, this estimate assumes linear behavior beyond the available time points, which may not fully capture the actual biological dynamics. Therefore, the DRB should be interpreted as an approximation.

### 3.2. Bio-Oss^®^

In Lambert’s study, the mean percentage of newly formed bone was 0.1%, 14.8%, and 16% at 1 week, 5 weeks, and 6 months, respectively. Concurrently, the residual graft decreased from 42.7% to 40.3% and then to 34.9% over the same time intervals (Table 1) [28]. At 6 months, the two lines remained far apart, suggesting that the processes of bone formation and graft resorption had plateaued without reaching convergence. rendering any extrapolation for the assessment of the break-even point both unjustified and unreliable (Figure 1A; Table 2).

In Yamada’s study, on the 14th and 84th days, the newly formed bone increased from 7.0% to 34.4%, while the residual graft decreased from 47.8% to 33.1% (Table 1) [29]. These values show an active regenerative process still in progress at the latest time point.

**Figure 2:**
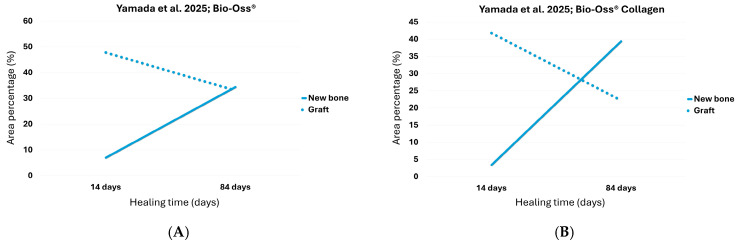
The point at which the two lines intersect represents the break-even point for (**A**) Bio-Oss^®^.; (**B**) Bio-Oss^®^ Collagen [29].

Linear extrapolation revealed that the break-even point was estimated at approximately 81.8 days, with both components representing 33.6% of the total evaluated tissue. This suggests that the turnover equilibrium was reached only marginally before the end of the observation period, indicating a slower dynamic compared to other groups (Figure 2A; Table 2).

### 3.3. Bio-Oss Collagen^®^

On the 14th and 84th days, the percentage of newly formed bone increased from 3.4% to 39.4%, while the residual graft decreased from 41.8% to 22.2% (Table 1) [29]. These values indicate substantial regenerative activity and biomaterial resorption over time.

Linear extrapolation of the trends estimated the break-even point at approximately 62.3 days, with both the new bone and residual graft reaching 28.3% of the total evaluated tissue.

These results suggest that the equilibrium between bone apposition and graft degradation occurred well before the endpoint of observation, confirming a sustained and efficient turnover dynamic.

### 3.4. Maxresorb^®^

At 2 and 10 weeks, the mean percentage of newly formed bone was 2.65% and 34.2%, respectively, whereas the residual graft decreased from 52.05% to 37.38% (Table 1) [30]. Although the trends clearly indicate an active regenerative process, the values had not equalized by the latest observation point.

**Figure 3:**
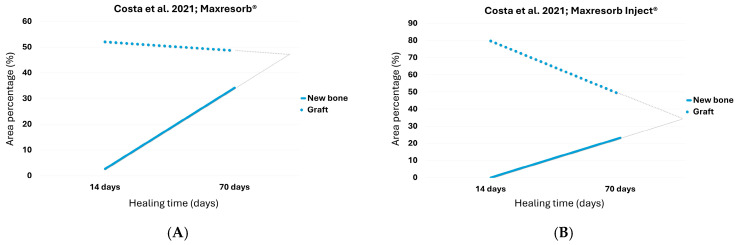
The point at which the two lines intersect represents the break-even point for (**A**) Maxresorb^®^ granules; (**B**) Maxresorb^®^ Inject [30].

Based on linear extrapolation between these two time points, the break-even point was estimated to occur at approximately 73.85 days, with both components reaching 36.37% of the total evaluated tissue (Figure 3A; Table 2).

Notably, this estimate assumes linear behavior beyond the last time point, which may not fully reflect the actual biological dynamics. Therefore, the break-even point should be interpreted as an approximation within the limits of available data.

### 3.5. Maxresorb^®^ Inject

At 2 and 10 weeks, the mean percentage of newly formed bone was 0.08% and 23.28%, respectively, while the residual graft decreased from 79.72% to 48.63% (Table 1) [30]. These opposing trends clearly reflect active bone regeneration and concurrent material resorption; however, they had not yet converged by the last observation point.

Based on linear extrapolation between the two time points, the break-even point was estimated to occur at approximately 96.1 days, with both components reaching 34.1% of the total evaluated tissue (Figure 3B; Table 2).

As in the previous case, this estimation assumes a linear progression beyond the observed interval and should therefore be interpreted as an approximation within the limits of the available data.

### 3.6. Gen-Os^®^

At 2 and 8 weeks, the percentage of newly formed bone increased from 6.17% to 26.72%, while the residual graft decreased from 35.2% to 9.62% (Table 1) [31]. These opposing trends indicated an active process of bone regeneration and biomaterial resorption.

**Figure 4:**
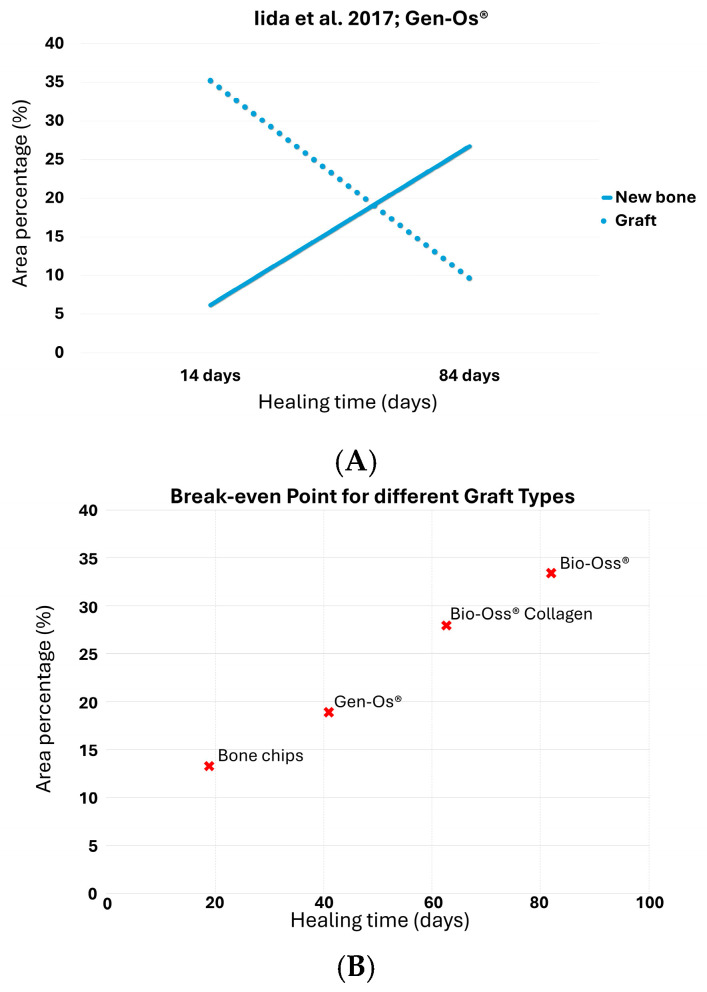
(**A**) The point at which the two lines intersect represents the break-even point forGen-Os^®^ [31]; (**B**) graphical comparison with the data obtained from the analysis of different graft types.

Linear extrapolation of the respective trends revealed that the break-even point was reached at approximately 40.4 days, with both components representing 19.1% of the total evaluated tissue (Figure 4A; Table 2).

This value suggests that the turnover process achieved equilibrium well before the final observation point, highlighting a relatively rapid replacement of the biomaterial by vital bone.

### 3.7. Risk of Bias Assessment

The overall risk of bias of the included studies was moderate. As commonly observed in preclinical animal research, some domains (e.g., allocation concealment, blinding of outcome assessment, random housing) were frequently reported insufficiently and therefore judged as “unclear risk.” By contrast, domains related to baseline characteristics, selective outcome reporting, and incomplete data were generally judged as “low risk.”

## 4. Discussion

In this study, we applied the break-even point to analyze the relationship between new bone formation and graft resorption of different types of biomaterials used for sinus floor elevation in rabbits. The results revealed varying patterns of bone formation relative to graft degradation, highlighting distinct healing dynamics among the different graft materials evaluated.

While static histomorphometric parameters such as BIC% have long been used to assess bone regeneration and implant integration, they offer only a partial snapshot of the complex biological processes involved. By contrast, the concept of Dynamic Regenerative Balance (DRB), introduced in the present work, aims to capture the biological equilibrium between bone formation and graft resorption, serving as a temporal and functional indicator of regenerative maturity. To approximate this concept quantitatively, we employed the break-even point, defined as the time and corresponding percentage at which newly formed bone equals the residual graft.

The results from the present study revealed that autogenous bone chips showed the most rapid progression, with the break-even point occurring at approximately 18.4 days [28]. This reflects a high remodeling capacity and near-complete graft resorption by the fifth week. This result is consistent with the known biological behavior of autografts. Nevertheless, no parameters have previously been used to define this phenomenon, whereas the break-even point offers a quantifiable approach to describe the dynamics of bone formation and graft resorption over time. Additionally, a graphical comparison with the data obtained from the analysis of all biomaterials makes these differences even more evident (Figure 4B).

Among the xenografts, Bio-Oss^®^ and Bio-Oss Collagen^®^ exhibited slower turnover, with break-even points estimated at 81.8 and 62.3 days, respectively [29]. The presence of collagen in the latter decreased the initial concentration and appears to have moderately accelerated the resorption profile.

However, in another of the studies included in this report, the break-even point with Bio-Oss^®^ was not reached even after six months of healing [28]. The lack of intersection between the lines at 6 months may indicate that both bone formation and graft resorption had already reached a steady state, and that no further convergence, and therefore no break-even point would be achieved with additional time. The differing outcome compared to the previously mentioned study [29] may be primarily due to a lower amount of new bone formation, even though the graft percentage was similar to that observed in that study. The discrepancy may be related to the different total amount of biomaterial used in the two studies or maybe to the orientation of the biopsy cuts, sagittal in Lambert’s study [28] and coronal in Yamada’s [29]. The sagittal sections were evidently taken through the center of the sinus, the region most distant from the bony walls, which are the major source of osteogenic activity. By contrast, Yamada’s study employed coronal sections, including only a portion of the central area. However, the data from these two studies suggest that the presence of non-resorbed biomaterial, despite its excellent osteoconductive properties [34], may not prevent but rather occupy space needed for new bone formation.

Maxresorb^®^ (biphasic calcium phosphate), in both granular and injectable formulations, exhibited slow remodeling dynamics, with estimated break-even points beyond 70 and 90 days, respectively [30]. This observation aligns with the well-documented slow resorption rate of BCP ceramics, particularly in dense granule or nanoparticulate formulations, where limited porosity and high hydroxyapatite content retards biodegradation and substitution by new bone [33,35,36,37]. Nevertheless, these estimations require validation with longer healing periods.

Gen-Os^®^, a collagenic porcine-derived material, reached the break-even point at approximately 40.4 days, suggesting a relatively faster remodeling rate compared to bovine xenografts [31]. This finding may be attributed to its partially preserved organic content and more favorable resorption characteristics, in contrast to Bio-Oss Collagen, which contains 10% porcine type I collagen added to the inorganic matrix.

Taken together, these results support the use of the break-even point as a complementary analytical tool capable of differentiating between materials not merely on the basis of bone gain or graft persistence, but on their synchrony, how well their resorption aligns with new bone formation. This temporal coherence is critical for regenerative procedures, especially in load-bearing contexts or in anatomical sites with limited remodeling capacity. By shifting the analytical focus from static values to dynamic equilibrium, the present approach may inform future material selection and protocol design. The break-even point does not replace traditional metrics such as BIC%, or BGC% (bone-to-graft contact percentage) [37], or bone volume fraction but rather enhances their interpretative value by introducing a temporal, regenerative perspective grounded in the broader conceptual framework of the Dynamic Regenerative Balance.

DRB represents a theoretical ideal, a concept referring to a dynamic moment of biological transition whereas the break-even point is its measurable approximation derived from experimental data. This relationship can be compared to that between the concept of osseointegration and its quantitative measure, BIC%. By applying this analytical model across different biomaterials, we were able to uncover substantial differences in regenerative kinetics that are not captured by conventional static metrics.

It should be considered that the break-even point, as proposed in the present article, was calculated using percentage values. Nevertheless, it can also be determined by using absolute measurements. While the resorbable nature of a biomaterial is generally considered a favorable property, it inevitably entails a reduction in graft volume, which may negatively influence the healing outcome. Thus, the resorbability of a biomaterial should be carefully considered when planning regenerative procedures.

This consideration is particularly relevant in sinus floor elevation procedures. Graft volume loss between different periods of healing has been reported in several experimental [27,38,39,40] and clinical studies [41,42,43,44]. Likewise, the studies included in the present analysis showed varying degrees of dimensional reduction related to the biomaterial used. Among the investigated materials, Bio-Oss exhibited the lowest dimensional reduction (~9%) [28,29], followed by Bio-Oss Collagen (~17%) [29]. By contrast, autogenous bone [28] and Gen-Oss [31] showed the highest resorption rates, with dimensional losses of approximately 42% after 5 weeks and 68.6% after 6 months for the autogenous bone, and 47% for Gen-Os. Maxresorb and Maxresorb Inject exhibited intermediate values, with reductions of 29.3% and 34% between 2 and 10 weeks of healing, respectively [30].

It should also be emphasized that the biological quality of regenerated bone differs depending on the amount of residual graft. While non-resorbable particles may secure long-term volume stability, they may result in a “hybrid bone” in which graft remnants persist within the regenerated tissue. From a biological perspective, complete replacement by vital bone is preferable, as it ensures a fully living matrix with higher regenerative potential. The DRB framework may therefore help to identify biomaterials that achieve a more favorable balance between dimensional stability and substitution by vital bone.

Beyond its quantitative role in bone biology, the Dynamic Regenerative Balance and the break-even point, understood as a moment of balance between formation and loss, resonates with the philosophical notion of “concept.” In medicine and dentistry, a concept is more than a measurable parameter; it frames understanding, guides practice and evolves over time. From a philosophical perspective, concepts are abstract representations that enable categorization and communication of complex phenomena (e.g., osseointegration), and their evolution has been described as part of broader shifts in scientific paradigms [45]. As Kuhn argued, scientific progress does not proceed only through incremental additions but also through paradigm shifts triggered by unexpected discoveries. Classic examples include Fleming’s observation of penicillin, and Brånemark’s identification of osseointegration, both initially serendipitous findings that ultimately reshaped entire fields, serendipity being defined as the occurrence of discoveries at the intersection of chance and wisdom [46].

As emphasized by Immanuel Kant, concepts are mental representations that unify objects under a single idea, enabling us to understand, categorize, and ultimately make knowledge possible. They mediate between empirical data and human understanding [47]. Similarly, Charles Sanders Peirce regarded concepts as inferential tools derived from pragmatic implications [48]. This perspective resonates with Lakoff and Johnson’s theory that many scientific ideas are structured through underlying metaphors, which shape the way we reason about complex systems [49]. In this regard, both the Dynamic Regenerative Balance and the break-even point can be seen as conceptual metaphors: they translate the biological transition between resorption and formation into an intelligible analytical framework, much as the break-even point has long served in economics to depict the transition between loss and profit.

In fact, the very possibility of considering the Dynamic Regenerative Balance and the break-even point as concepts emerges through the metaphor of a dynamic and temporal point of equilibrium, transforming a complex biological process into a conceptual model that is accessible and applicable.

It is worth reiterating that the break-even point has already been applied in previous studies to investigate the dynamics of osseointegration and peri-implant bone density across a variety of preclinical models, including humans, dogs, and rabbits [25,26]. These studies have shown that this parameter is sensitive to key variables such as species, implant surface characteristics, bone type (cortical vs. trabecular), loading protocols, and immediate implant placement into extraction sockets. In the context of sinus augmentation procedures, it has proven capable of detecting localized differences in regenerative behavior, particularly between areas adjacent to the sinus walls and those beneath the sinus mucosa [27]. The present analysis illustrates its value in evaluating the balance between new bone formation and biomaterial resorption, providing a clear indication of the substitution potential of different grafts. Additional applications could include alveolar ridge preservation procedures, bone formation in post-extraction sockets, guided bone regeneration of peri-implant defects, vertical and horizontal ridge augmentation, and even periodontal regenerative therapies. In particular, rabbit calvarial critical size defects may represent an advantageous model for testing this approach, since they provide standardized conditions without the additional variables introduced by sinus augmentation. In such settings, the break-even point could allow a more direct comparison among scaffolds and biomaterials, highlighting their relative ability to be resorbed while supporting new bone formation.

From a clinical perspective, understanding the dynamics of bone remodeling provides information that goes beyond volume stability alone. Materials that maintain volume but remain largely unresorbed may ensure dimensional integrity but result in hybrid bone, whereas materials that reach the break-even point earlier may favor a more complete replacement by vital bone. Such information can guide clinicians in material selection depending on whether long-term stability or biological quality of the regenerated tissue is prioritized. Moreover, identifying the approximate timing of the break-even point may also assist in planning implant placement or loading protocols, aligning clinical decisions with the biological dynamics of regeneration.

These prior findings, together with the present analysis, underscore the versatility and robustness of the break-even point as a tool for capturing dynamic patterns of healing that would otherwise remain hidden using conventional static metrics.

**Figure 5:**
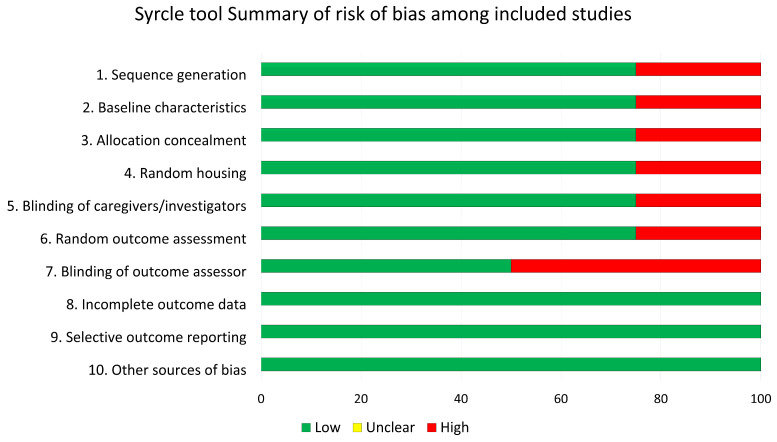
Summary of the risk of bias assessment using the SYRCLE’s tool. Green = low risk; yellow = unclear risk; red = high risk.

The risk of bias analysis (Figure 5) showed that our studies were generally at low risk, with the exception of one experiment where allocation could be identified at histology. By contrast, the external study by Lambert et al. presented a high risk in several domains. Although this tool is more often applied in systematic reviews, we included it here to enhance transparency regarding methodological quality.

An important limitation of the present analysis lies in the heterogeneity of the included studies, particularly regarding healing intervals, biopsy orientation, and the amount of grafted material. In addition, the calculation of the break-even point was based on the assumption of linear progression between two observation periods, which implies that the closer the interval is to the actual break-even point, the more reliable the estimate. Moreover, it should be emphasized that bone healing and remodeling are complex, non-linear processes characterized by variable phases of acceleration and plateau.

The assumption of linearity was therefore adopted only as a practical approximation to allow the estimation of the break-even point from limited data. This simplification should not be interpreted as a literal representation of the biological process but rather as a methodological tool to extract indicative trends from the available heterogeneous data. Evidence from some of the included studies, such as Lambert et al. (2011) [28] and Iida et al. (2017) [31], which examined three different healing intervals, clearly shows that bone formation and graft resorption do not follow a strictly linear course but instead progress through phases of rapid change followed by relative stabilization. These observations reinforce that the linear interpolation adopted in the present analysis is only a pragmatic approximation, while the underlying biological processes remain more complex. Consequently, the reported values should be regarded as illustrative examples rather than definitive benchmarks.

It should also be emphasized that this work is not a systematic review, but a methodological study designed to demonstrate the applicability of the break-even point. Only four articles were included because they provided the specific type of data required (i.e., new bone formation and residual graft values at more than one healing interval). In particular, the inclusion of Lambert et al. was necessary to represent autogenous bone, since no suitable study from our own group was available on this material. A larger and more diverse body of evidence will be required to establish representative averages and to validate the method in a systematic manner.

Moreover, the present analysis already provides an initial indication of the most appropriate time frames for evaluating the break-even point. For example, in the case of Gen-Os^®^, the break-even point was located within the available interval, suggesting that future studies should include intermediate time points to narrow this range. Conversely, for Maxresorb^®^, the break-even point was not reached within the observed period, indicating that longer follow-up times are necessary. Such adjustments in study design may help to more precisely identify the dynamics of graft resorption and new bone formation for different biomaterials.

Finally, while the present analysis offers preliminary indications of the time frames in which the break-even point may occur for different biomaterials, identifying the most suitable and generalizable intervals would require a specifically designed experimental study involving several animals, multiple grafting materials, and standardized methodologies. Alternatively, a systematic review could be conducted, although such an approach would inevitably result in the inclusion of heterogeneous studies, thus limiting the precision of the recommendation.

## 5. Conclusions

By calculating the time point at which new bone formation equals graft resorption, the break-even point provides insight into the temporal dynamics of healing and represents a valuable complement to static histomorphometric metrics such as BIC% or bone volume fraction. The present analysis illustrated how different biomaterials may present distinct break-even behaviors, with Gen-Os^®^ showing values within the observed interval and Maxresorb^®^ and Bio-Oss^®^ requiring longer observation periods. These results, although based on a limited number of studies, demonstrate the potential of the break-even point to capture the dynamic balance between tissue formation and degradation. As an operational expression of the broader concept of Dynamic Regenerative Balance, the break-even point may serve as a useful analytical framework to compare biomaterials and to guide the design of future experimental and clinical studies aimed at validating and refining this approach. From a clinical perspective, such knowledge may help in selecting biomaterials according to the desired balance between dimensional stability and substitution by vital bone, as well as in planning the timing of implant placement and loading protocols.

## Figures and Tables

**Table 1:** Overview of the analyzed articles, graft materials, and healing periods. Density percentages of bone and graft were evaluated at two different timepoints: T1 and T2.

Authors	Graft	Periods Analyzed	T1		T2	
			% Bone	% Graft	% Bone	% Graft
Lambert et al. [28]	Autogenous	1 week–5 weeks	0.3	22.7	32.7	0.0
	Bio-Oss	1 week–6 months	0.1	42.7	16	34.9
Yamada et al. [29]	Bio-Oss	2 weeks–12 weeks	7.0	47.8	34.4	33.1
	Bio-Oss Collagen	2 weeks–12 weeks	3.4	41.8	39.4	22.2
Costa et al. [30]	HA-βTCP granules	2 weeks–10 weeks	2.65	52.05	34.2	37.38
	HA-βTCP paste	2 weeks–10 weeks	0.08	79.72	23.28	48.63
Iida et al. [31]	Gen-Os	2 weeks–8 weeks	6.17	35.2	26.72	9.62

**Table 2:** Break-even points values evaluated based on percentage of bone and residual graft at two timepoints and expressed in Days and BIC %. The loss of dimension is also reported. ^a^, the lines had not yet converged by the last observation point so that the estimation assumes linear behavior beyond the last time point.

Authors	Graft	Periods Analyzed	Days	BIC%	Dimension Loss Between Periods
Lambert et al. [28]	Autogenous	1 week–5 weeks	18.4	13.5	42.4% (68.6% after 6 months)
	Bio-Oss	1 week–6 months	NA	NA	9.4%
Yamada et al. [29]	Bio-Oss Collagen	2 weeks–12 weeks	62.3	28.3	16.8%
	Bio-Oss	2 weeks–12 weeks	81.8	33.6	8.9%
Costa et al. [30]	HA-βTCP granules	2 weeks–10 weeks	73.85 ^a^	36.37 ^a^	29.3%
	HA-βTCP paste	2 weeks–10 weeks	96.1 ^a^	34.1 ^a^	34.0%
Iida et al. [31]	Gen-Os	2 weeks–8 weeks	40.4	19.1	47.0%

## Data Availability

The raw data supporting the conclusions of this article will be made available by the authors on request.

## Supplementary Materials

The following supporting information can be downloaded at https://www.mdpi.com/article/10.3390/dj13100469/s1, S1: Break-even point calculator.xlsx.
